# Migration of a Hem-O-Lok clip into the cecal lumen following laparoscopic appendectomy: a case report

**DOI:** 10.1186/s12876-026-04841-2

**Published:** 2026-05-07

**Authors:** Runjie Hou, Jing Guo, Mingyue Du, Zhilong Zhang, Jijun Wang

**Affiliations:** 1 Department of General Surgery, Baotou Central Hospital, Baotou, China; 2 Central Clinical Medical College, Baotou Medical College, Baotou, China; 3 Department of General Surgery, Xiwu Banner People’s Hospital, Xilinhot, China; 4 The 969th Hospital of the Joint Logistics Support Force of the Chinese People’s Liberation Army, Hohhot, China

**Keywords:** Appendectomy, Surgical Instruments, Foreign-Body Migration, Colonoscopy, Case Report

## Abstract

**Background:**

Hem-O-Lok clips are widely used in laparoscopic appendectomy (LA). Although postoperative clip migration is rare, it is a clinically significant complication. Previous reports have mainly focused on clip migration after cholecystectomy and urologic surgery. To the best of our knowledge, there have been no reports of clip migration into the intestinal lumen after LA. This report suggests that such migration may occur after LA, highlights an important potential device-related complication, and adds to the relevant literature.

**Case presentation:**

A 44-year-old male experienced persistent dull right lower abdominal pain for three days, two months after LA. Physical examination showed tenderness at McBurney’s point, while laboratory tests revealed no abnormalities. The patient had received two Hem-O-Lok clips during surgery two months prior. Abdominal computed tomography (CT) revealed a high-density shadow in the ileocecal region. Colonoscopy confirmed a Hem-O-Lok clip embedded at the appendiceal orifice within the cecal lumen. The patient refused endoscopic removal, opting for conservative observation. At the six-month follow-up, the patient’s pain had completely resolved.

**Clinical discussion:**

The mechanism of this complication remains unclear and may involve the combined effects of local inflammation and foreign body-related reaction. CT may serve as an initial screening tool, and endoscopy is helpful for establishing the diagnosis. As there is currently no standardized treatment strategy, conservative observation, endoscopic intervention, and surgical treatment may all be considered as options, depending on the individual situation. In clinical practice, attention should also be paid to the standardized use of clips in order to reduce the risk of similar complications.

**Conclusion:**

This case highlights the potential risk of Hem-O-Lok clip migration after LA. Clinicians should increase their awareness of this rare complication.

## Background

Acute appendicitis ranks among the most common surgical emergencies globally [1]. LA is currently recognized by major international guidelines as the gold-standard surgical approach for acute appendicitis and has been widely implemented worldwide [2]. A crucial step in LA is the secure closure of the appendiceal stump. Due to its operational simplicity and reliable closure properties, the Hem-O-Lok clip has become one of the preferred choices for appendiceal stump closure during LA [3]. However, as an implantable foreign body, the Hem-O-Lok clip poses a rare but real risk of postoperative migration. The existing literature on this complication is limited, with most reports focusing on laparoscopic cholecystectomy and urological surgery [4–6]. Among these reports, cases of migration into the intestinal lumen are extremely rare, with the majority involving displacement into the duodenum [7, 8]. To date, only one case following LA has been reported, in which the clip detached and migrated into the fallopian tube [9]. To our knowledge, there have been no documented cases of a clip migrating directly from its original site on the appendiceal stump into the cecal lumen.

Therefore, this paper reports a rare device-related complication and discusses its possible mechanism, diagnostic approach, management strategy, and preventive considerations in conjunction with the relevant literature. With the increasing popularity of laparoscopic techniques and the widespread clinical use of Hem-O-Lok clips, this case aims to improve surgeons’ awareness of this type of complication and provide a reference for the clinical recognition and management of similar cases. This case report was prepared in accordance with the CARE (CAse REport) checklist [10].

## Case presentation

**Fig. 1:**
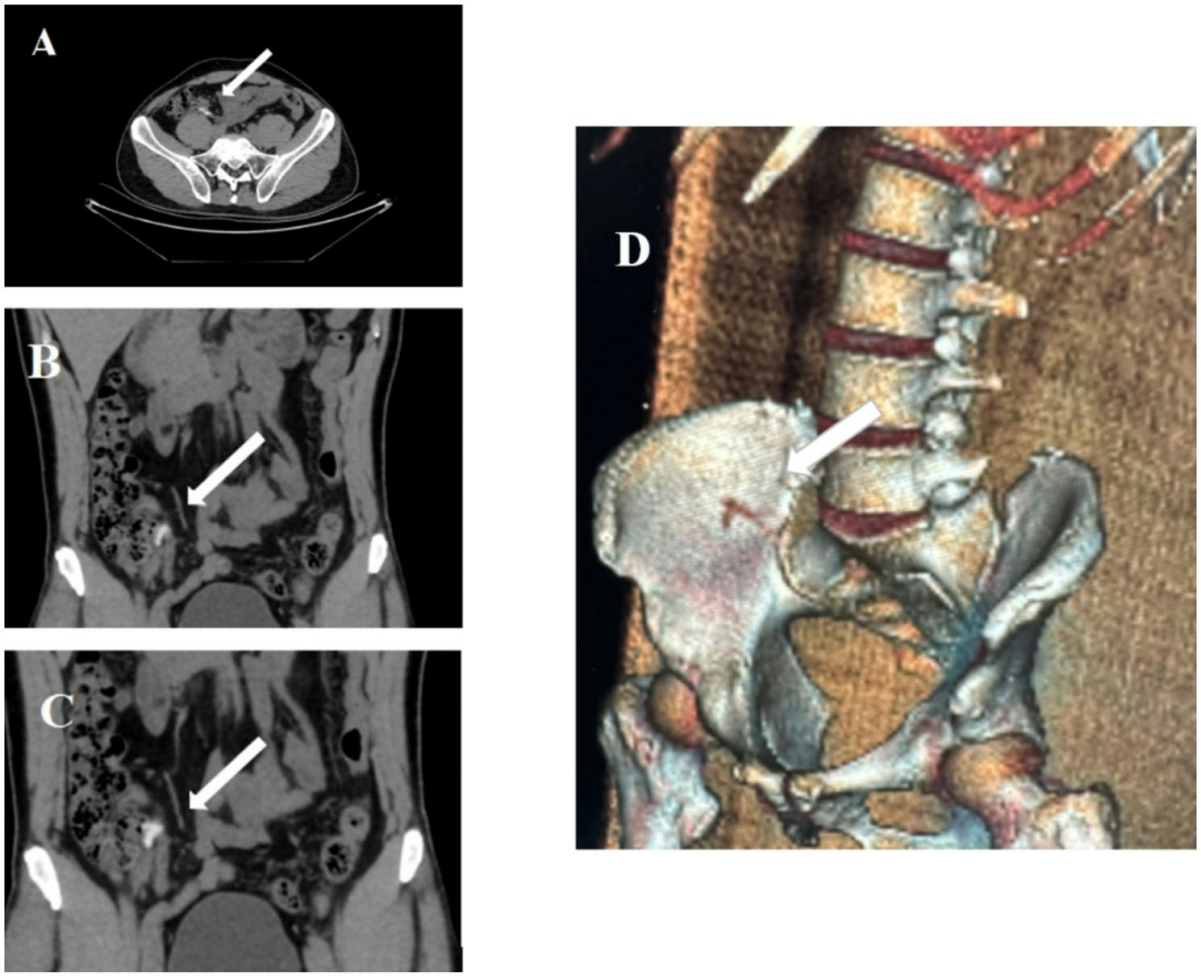
Abdominal CT images and 3D reconstruction showing the suspected Hem-O-Lok clips used during laparoscopic appendectomy (**A**, axial view; **B **and **C**, coronal views; **D**, 3D reconstruction), with arrows indicating the suspected clips

**Fig. 2:**
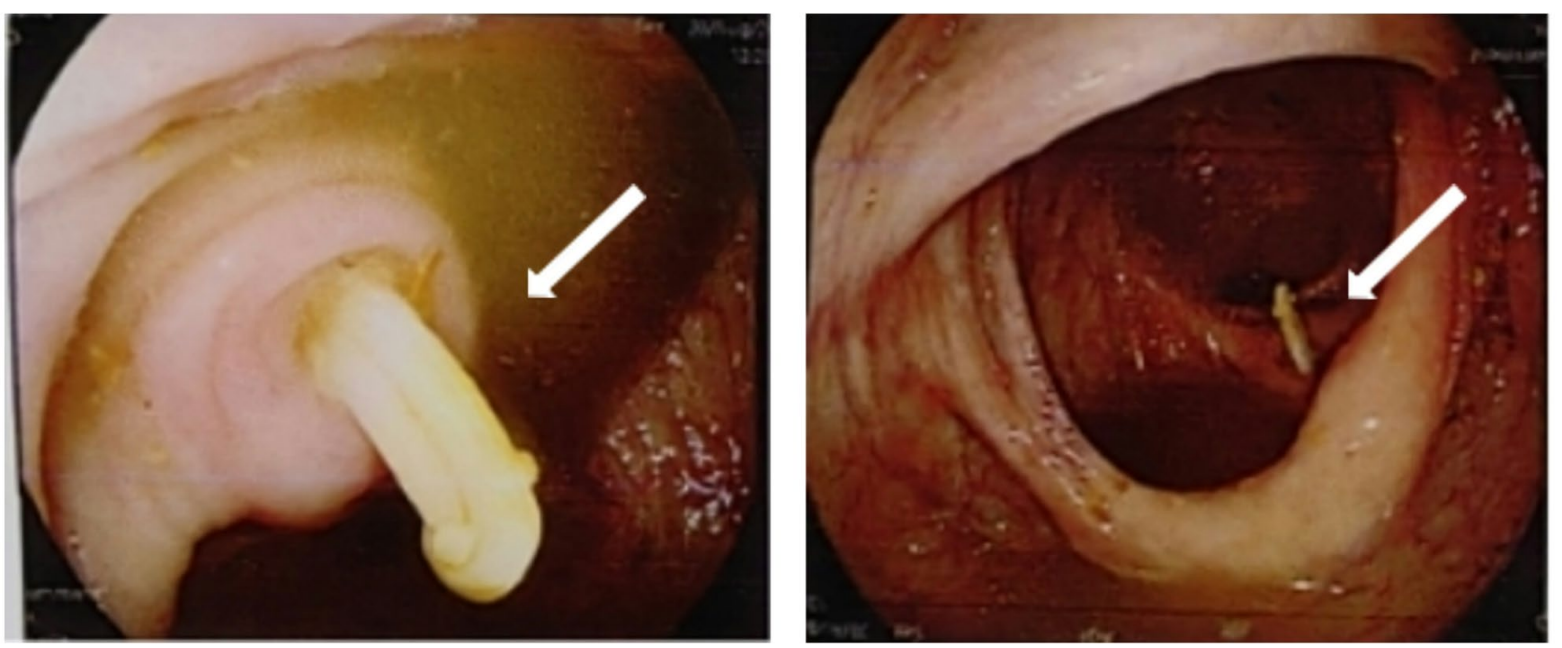
Colonoscopy showing a Hem-O-Lok clip at the appendiceal orifice, partially embedded in the bowel wall (arrows)

A 44-year-old male visited our gastrointestinal surgery outpatient clinic, complaining of persistent dull pain in the right lower abdomen for three days. He had undergone LA two months earlier. Physical examination revealed tenderness at McBurney’s point. Laboratory tests yielded the following results: hemoglobin at 145.00 g/L, white blood cell count (WBC) of 7.53 × 10⁹/L, and neutrophil percentage of 53%. All laboratory results were within normal limits. Subsequent abdominal CT revealed a high-density shadow in the ileocecal region (Fig. 1). Further inquiry into the medical history and review of the patient’s previous hospitalization records and operative notes showed that he had suffered appendicitis 9 months earlier, which had been managed conservatively with antibiotics. Two months before the current visit, he underwent LA because of recurrent acute appendicitis. Preoperative CT showed mild enlargement of the appendix, with a diameter of approximately 12 mm, and no fecalith was identified. A standard three-port laparoscopic appendectomy was performed. Intraoperatively, the appendix was identified along the right colon. The appendix showed marked congestion and edema and was adherent to the surrounding tissues, but no perforation was observed. After adhesiolysis around the appendix, one Hem-O-Lok clip (KANGJI, Hangzhou Kangji Medical Instruments Co., Ltd.; model: KJ-JZJ04L, large size) was used to ligate the mesoappendiceal vessels. The mesoappendix was then divided with electrocautery and carefully dissected to the appendiceal base. Another Hem-O-Lok clip of the same model was applied approximately 0.5 cm from the appendiceal base to close the appendiceal stump, after which the appendix was resected. No purse-string inversion suture was performed. Subsequent colonoscopy confirmed that a Hem-O-Lok clip was impacted at the appendiceal orifice within the cecal lumen (Fig. 2). Based on the previous surgical history and colonoscopic findings, the diagnosis of Hem-O-Lok clip migration into the cecal lumen after LA was established. Endoscopic submucosal dissection (ESD) was planned for clip removal. However, after discussing the potential risk of perforation with the patient’s family, they declined the procedure. Considering the lack of consent and the possibility of spontaneous clip expulsion, a conservative observation strategy with close follow-up was adopted, with instructions to seek immediate medical attention if pain worsened. At the six-month follow-up after colonoscopy, the patient reported that his abdominal pain had completely resolved and declined a repeat colonoscopy.

## Discussion

Acute appendicitis is a prevalent acute abdominal condition. Studies indicate there were about 17 million new cases of appendicitis worldwide in 2021 [1]. Current guidelines endorse LA as the preferred treatment for acute appendicitis [2]. The crux of LA lies in the secure and effective closure of the appendiceal stump. Safe closure techniques for the appendiceal base include suturing, non-absorbable polymer clips (e.g., Hem-O-Lok), endoloops, endostaplers, and metal endoclips [3, 11, 12]. Studies suggest that Hem-O-Lok clips are cost-effective and can significantly shorten operative time, with their reliability for stump closure being comparable to other methods [3]. Consequently, it has become one of the preferred options for stump closure in LA [3]. Due to their convenience, these clips are also widely used in laparoscopic cholecystectomy [5, 7], urological surgeries [4, 6], and video-assisted thoracoscopic surgery (VATS) [13]. However, postoperative migration of Hem-O-Lok clips is a rare but noteworthy complication.

A PubMed database search using the keywords “Hem-O-Lok” and “migration” (without restrictions on publication date or language) revealed that existing reports mainly focus on laparoscopic cholecystectomy and urological surgeries [4–8]. Only one case of clip migration after LA has been reported [9], where the clip migrated to the fallopian tube. Its migration route, diagnosis, and treatment strategy likely differ greatly from the present case, limiting its referential value.

The exact mechanism of a Hem-O-Lok clip migrating into the intestinal lumen remains unclear. Since there have been no prior reports of a clip entering the cecal lumen via the appendiceal stump after LA, we can only refer to related cases of clip migration into the duodenal lumen after cholecystectomy or into anastomotic sites after distal gastrectomy [7, 8, 14]. We hypothesize that in this case, the mechanism may involve two factors: first, appendicitis-related inflammation may involve the appendiceal base and adjacent cecal tissue, resulting in increased tissue fragility and possibly post-inflammatory scar formation with reduced tissue elasticity, thereby creating conditions that may facilitate clip migration; second, as a foreign body, the Hem-O-Lok clip may provoke a sustained local inflammatory reaction, and chronic inflammatory stimulation may be associated with local tissue erosion or defect formation, thereby promoting gradual migration of the clip into the intestinal lumen. It should be emphasized that no histopathological evidence from the appendiceal stump or surrounding tissue was available in this case. Therefore, the proposed explanation remains a theoretical inference based on the clinical presentation and previous literature rather than a histologically confirmed mechanism.

Based on previous reports of Hem-O-Lok clip migration into the intestinal lumen and our experience in managing the present case [7, 14], we provide reference diagnostic and therapeutic strategies. From a diagnostic perspective, in patients with persistent localized abdominal pain after LA, a detailed surgical history, particularly the use of clips, is the primary clue. Abdominal CT can serve as an initial screening tool to detect abnormal high-density shadows in the ileocecal region. Previous studies have shown that Hem-O-Lok clips may appear as visible high-density objects on CT, with a density of approximately 222–223 HU [15]. Colonoscopy can provide a definitive diagnosis by directly visualizing the Hem-O-Lok clip and evaluating the degree of embedment and the surrounding mucosal condition. Before penetrating the intestinal wall, the clip may appear endoscopically as a submucosal tumor (SMT), which should be differentiated from polyps, cysts, and stromal tumors to avoid overtreatment [7].

**Fig. 3:**
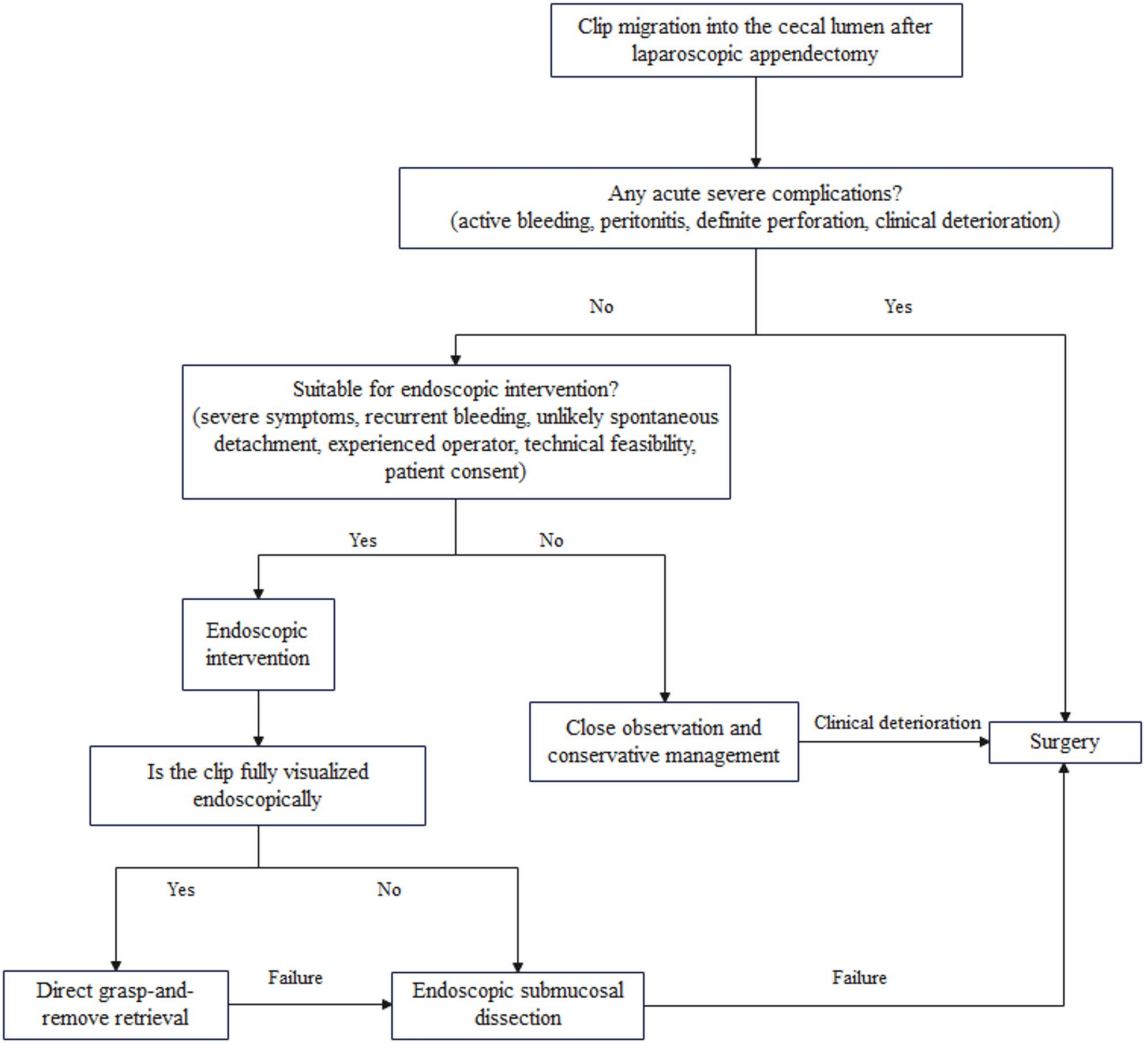
A reference management algorithm for Hem-O-Lok clip migration into the cecal lumen after laparoscopic appendectomy

With regard to treatment, previous reports show that the management of clips migrating into the intestinal lumen is not uniform and includes endoscopic intervention (direct retrieval or ESD-assisted management) and conservative observation, with some cases undergoing spontaneous separation and expulsion [14, 16, 17]. However, cases successfully removed by direct endoscopic grasping with forceps have mainly involved migration into the duodenum [7, 18], where the degree of clip exposure can usually be observed relatively completely under endoscopy. In contrast, in the present case, the clip was partially impacted and covered by tissue around the appendiceal orifice, and direct retrieval might have increased the risk of local mucosal tearing or even perforation. At the same time, reports of successful ESD treatment of migrated clips have involved migration into the rectum [16], and that experience cannot be simply extrapolated to the appendiceal orifice or cecal region. Previous studies have shown that severe tissue fibrosis and right-sided colonic location are both associated with an increased risk of perforation during ESD [19]. Although some studies have suggested that ESD is feasible for lesions involving the appendiceal orifice after appendectomy, such procedures require considerable operator experience and are associated with relatively high risks of bleeding and perforation [20, 21]. Therefore, for cases of clip migration near the appendiceal orifice and possibly associated with post-inflammatory scarring or fibrosis, ESD may not be regarded as the routine first-line option. Based on the present case and the available literature, the following management pathway is provided for reference in patients with Hem-O-Lok clip migration into the cecal lumen after LA (Fig. 3): for patients with acute severe complications, such as active bleeding, definite perforation, or peritonitis, surgical treatment should be given promptly; for patients with persistent prominent symptoms, recurrent bleeding, or a low likelihood of spontaneous detachment, endoscopic intervention may be considered cautiously after full evaluation, with surgical treatment if necessary; and for patients with mild symptoms, no active bleeding, no peritonitis or definite evidence of perforation, and refusal of invasive treatment, conservative observation with close follow-up may be a reasonable option. It should also be noted that proton pump inhibitors (PPIs) were used in some previous cases managed conservatively [14, 17], but this approach was mainly reported in cases in which the migration endpoint was the duodenum. The main mechanism of PPIs is suppression of gastric acid secretion; therefore, their mucosal protective effect is mainly relevant to the upper gastrointestinal tract, particularly the stomach and duodenum [22]. However, PPIs are unlikely to provide meaningful mucosal protection in the lower gastrointestinal tract [22, 23]. Therefore, routine use of PPIs during conservative management may not be necessary in patients whose clip migration endpoint is the colon.

In the present case, the main manifestation was dull pain in the right lower abdomen, without evidence of active bleeding, anemia, peritonitis, or other acute severe complications. In addition, the clip was partially embedded in the bowel wall on colonoscopy, and direct traction removal could have increased the risk of local mucosal tearing or even perforation. ESD also carries risks such as perforation and bleeding. Taking into account the lesion location, the potential procedural risks, and the patient’s refusal of invasive treatment, we ultimately chose conservative observation with close follow-up.

Based on this case and the previous literature, the following preventive considerations are provided for reference [14, 18]: any free or accidentally dislodged clips encountered during surgery should be removed promptly; for patients with marked inflammation, edema, or tissue fragility at the appendiceal base, the suitability of polymer clips for stump closure should be carefully evaluated, and alternative methods such as suturing or stapling may be considered when necessary; if Hem-O-Lok clips are still used to close the stump, they should be placed in a manner that avoids being too close to the cecal wall, while preserving an appropriate safe stump length. In clinical practice, a stump length of approximately 0.5 cm may be referenced to reduce the risk of stump appendicitis [24]. At the same time, the number of Hem-O-Lok clips should be minimized as much as possible to reduce local clip stacking, sustained compression, and foreign body irritation at the stump. Finally, the stability of stump closure and the condition of the surrounding tissues should be carefully checked at the end of the procedure.

Given that the migration endpoint is located within the cecal lumen and may become impacted at the appendiceal orifice, both the diagnosis and management of this complication may be more complex than those of other types of clip migration. With the widespread use of Hem-O-Lok clips, surgeons should increase their awareness of this type of device-related complication. In patients with persistent or recurrent right lower abdominal discomfort after surgery, this possibility should be included in the differential diagnosis after common causes such as stump inflammation and scar traction have been excluded.

## Conclusion

This case suggests that Hem-O-Lok clips may migrate into the cecal lumen after laparoscopic appendectomy. In patients with persistent postoperative abdominal pain, this rare device-related complication should be considered after common postoperative complications have been excluded. Its mechanism remains unclear. Diagnosis should be based on a comprehensive assessment of the surgical history, CT findings, and endoscopic examination, while treatment should be individualized according to the specific clinical circumstances. In addition, attention should be paid to proper appendiceal stump management and rational clip use in order to reduce the risk of similar complications.

## Data Availability

The data supporting the conclusions of this study are included in this published article. Due to patient privacy concerns, the raw data are not publicly available; de-identified data may be made available from the corresponding author upon reasonable request.

## Abbreviations

LA: Laparoscopic appendectomy
CT: Computed tomography
WBC: White blood cell count
ESD: Endoscopic submucosal dissection
SMT: Submucosal tumor
PPI: Proton pump inhibitor
VATS: Video-assisted thoracoscopic surgery
